# A Hybrid Bipolar Active Charge Balancing Technique with Adaptive Electrode Tissue Interface (ETI) Impedance Variations for Facial Paralysis Patients †

**DOI:** 10.3390/s22051756

**Published:** 2022-02-23

**Authors:** Ganesh Lakshmana Kumar Moganti, V. N. Siva Praneeth, Siva Rama Krishna Vanjari

**Affiliations:** 1 Department of Electrical Engineering, Indian Institute of Technology (IIT) Hyderabad, Kandi 502285, India; ganesh.moganti@gmail.com; 2 School of Electronics Engineering, VIT-AP University, Amaravati 522237, India; praneeth.18bev7022@vitap.ac.in

**Keywords:** functional electrical stimulation, electrode tissue interface impedance, active charge balancing, electrode shorting, pulse insertion and pulse modulation

## Abstract

Functional electrical stimulation (FES) is a safe, effective, and general approach for treating various neurological disorders. However, in the case of FES usage for implantable applications, charge balancing is a significant challenge due to variations in the fabrication process and electrode tissue interface (ETI) impedance. In general, an active charge balancing approach is being used for this purpose, which has limitations of additional power consumption for residual voltage calibration and undesired neurological responses. To overcome these limitations, this paper presents a reconfigurable calibration circuit to address both ETI variations and charge balancing issues. This reconfigurable calibration circuit works in two modes: An impedance measurement mode (IMM) for treating ETI variations and a hybrid charge balancing mode (HCBM) for handling charge balance issues. The IMM predicts the desired stimulation currents by measuring the ETI. The HCBM is a hybrid combination of electrode shorting, offset regulation, and pulse modulation that takes the best features of each of these techniques and applies them in appropriate situations. From the results, it is proved that the proposed IMM configuration and HCBM configuration have an optimal power consumption of less than 44 μW with a power ratio ranging from 1.74 to 5.5 percent when compared to conventional approaches.

## 1. Introduction

In the U.S. and European countries, facial paralysis is a common problem caused mainly due to the dysfunction of the seventh cranial nerve, which leads to loss of control on essential facial expressions (blinking, smiling, etc.) [1,2,3,4]. While 70–80% of people recover within a few months, others suffer chronic effects and severe lifelong dysfunction [5,6]. One of the major consequences of this paralysis is the loss of reflex and voluntary blink control, resulting in permanent corneal injury from ulceration or infection. Traditional approaches such as surgical [7,8,9] and mechanical [10,11], are used to address this problem, however they have significant drawbacks in terms of visibility, cosmetic appearance, implantation difficulties, patient safety, and inconvenience. Moreover, one’s face appearance becomes changed. Modern non-surgical ways to regain the functionality of paralyzed muscles have been introduced, such as magnetic stimulation, electro-chemical stimulation, and functional electrical stimulation (FES). Among these, FES is the most widely-used technique of injecting electrical pulses to the paralyzed muscles to regain its muscular moment. The FES approach creates neuronal response regeneration by using a bi-phasic waveform. In a bi-phasic waveform, a negative pulse is delivered first to generate an action potential, followed by a positive pulse to nullify the prior charge, resulting in an net charge to be zero.

In general, the charge created by a negative pulse is 1% to 5% greater or less than the charge induced by a positive pulse. This is due to the device irregularities that occur during fabrication, which are unavoidable. There are two key approaches, such as the passive charge balancing approach and active charge balancing approach that are evolved to address the charge balancing issues. Passive charge balancing approaches do not measure residual voltage, but simply eliminate it using various procedures, such as electrode shorting [12], connecting a DC blocking capacitor [13] series to the load, etc. However, as the DC blocking capacitor occupies a huge area, it is not suitable for implantable applications. In the electrode shorting approach, the electrodes are connected to the ground often for residual voltage discharge. As residual voltage discharge is dependent on the electrode time constant, this approach is limited to lower residual voltages (<25 mV).

On the other side, active charge balancing is a method of calculating the residual voltage and nullifying by various techniques such as offset regulation [14], pulse modulation [15], etc. In offset regulation, residual voltage is measured and nullified by applying an equal and opposite charge. However, this method produces undesired neurological responses at high residual voltages. As a result, this approach is limited to residual voltages ranging from low (<25 mV) to moderate (<50 mV). In pulse modulation, residual voltage is measured and nullified by applying equivalent opposite voltage to the next stimulation cycle. This approach efficiently works in high residual voltages (>50 mV). However, these two approaches consume extra power to nullify the residual voltages. In [16], we propose a novel architecture that measures the residual voltage and compares it with six predefined voltage levels. Based on the nearest levels, the opposite polar voltage is applied at load. However, it is focused on charge balancing issues and does not consider the ETI issue. It is not suitable for high residual voltage because it causes an undesirable neurological response. To address the above mentioned issues, this paper proposed a reconfigurable calibration circuit that works in both impedance measurement mode (IMM) and hybrid charge balancing mode (HCBM) configurations. The IMM is capable of predicting the desired stimulation currents by observing ETI variations. The HCBM is capable of achieving charge balancing with optimal power consumption. It is also a hybrid of electrode shorting, offset regulation, and pulse modulation that uses the best features of each technique and applies them in appropriate situations.

## 2. Proposed Architecture

**Figure 1:**
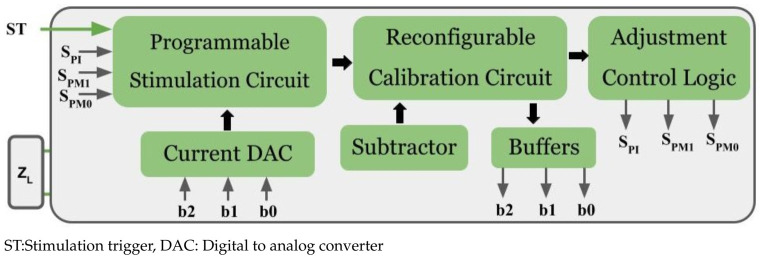
Proposed architecture.

The proposed architecture contains a programmable stimulation circuit, a current digital to analog converter (DAC), a reconfigurable calibration circuit, a subtractor, and adjustment logic as shown in Figure 1. This proposed architecture operates in three modes: IMM, stimulation mode (SM), and HCBM. Prior to stimulation, IMM is initiated, followed by stimulation mode. In addition, the hybrid charge balancing mode operates between each stimulation cycle. There are three phases in stimulation.

- Phase-1:Before stimulation, ETI impedance is calculated and adjusts the stimulation current (in IMM mode). Different patients require different stimulation current to excite the eyelid muscles.
- Phase-2: Adjusted stimulation current applied in the form of a biphasic waveform (in stimulation mode).
- Phase-3: After stimulation, residual voltage is calculated and nullified/reduced to a safe level (in HCBM mode). If the residual voltage is greater than 100 mV, it leads to tissue damage. Therefore, it is necessary to maintain the residual voltage within safety limits (<100 mV).

## 3. Impedance Measuring Mode (IMM)

The primary goal of IMM mode is to adjust the stimulation current by estimating ETI impedance. ETI impedance is derived using the ETI time constant. A high time constant indicates a high impedance, whereas a low time constant indicates a low impedance.

**Figure 2:**
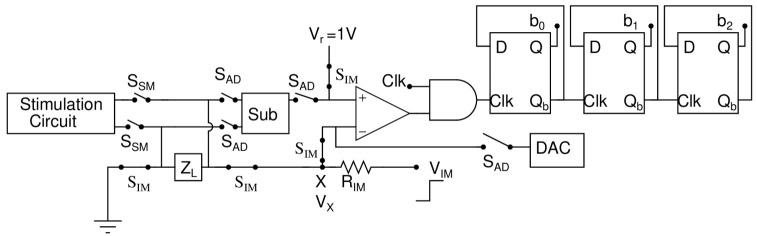
Circuit configuration of impedance measuring mode (IMM).

**Figure 3:**
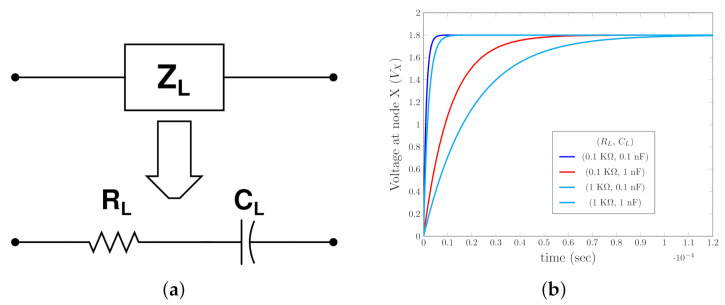
Electrode tissue interface (ETI) impedance. (**a**) ETI impedance equivalent circuit. (**b**) Voltage response at X node for different load impedance.

The circuit configuration of the impedance measuring unit is shown in Figure 2. In this mode, the $S_{IM}$ switches are turned ON while the rest of the switches are turned OFF. Initially, the step voltage ($V_{IM}$) is applied to the load ($Z_{L}$) through resistance ($R_{IM}$). As $Z_{L}$ is a combination of the series resistance and capacitor as shown in Figure 3a. Figure 3b shows the voltage waveform for different load impedance. Voltage ($V_{X}$) at node X is crucial for determining the time constant of $Z_{L}$. When $V_{X}$ is less than $V_{r}$, the comparator output is high and the AND gate produces clocks which are fed to the 3-bit counter. When $V_{X}$ is greater than $V_{r}$ then the comparator output is low and the AND gate halts producing the clock pulse to counter, which leads to a stored counter value in a 3-bit latch.

## 4. Calculation of ETI Impedance in IMM Mode

The following are the preliminary assumptions made before calculation:

- The ETI impedance is assumed as the resistor ($R_{L}$) series with capacitor ($C_{L}$).
- The capacitor ($C_{L}$) is uncharged, i.e., the initial load voltage, $V_{L}\left(0^{-}\right)$, is 0.
- The clock pulse is applied to an input of the AND gate.

When the step response is applied to the ETI, the voltage ($V_{X}\left(t\right)$) at node X can be expressed as:

(1) $$V_{X}\left(t\right)=V_{s}\left(1-e^{-t/\tau }\right)$$

where $V_{s}$ is the amplitude of the step voltage and τ is the time constant of the charging path. The τ can be expressed as:

(2) $$\tau =\left(R_{M}+R_{L}+R_{SW}\right)C_{L}$$

where $R_{M}$ is the measuring resistance and $R_{sw}$ is the switch resistance. The $V_{X}$ is compared with $V_{r}$ then the comparator output can be expressed as:

(3) $$V_{comp}\left(t\right)=\;\left\{\begin{array}{cc} Low & V_{X}\left(t\right)>V_{r}\\ High & V_{X}\left(t\right)<V_{r}. \end{array}\right.$$

The AND gate generates clock pulses until the comparator output remains low. The number of clock pulses generated by the AND gate is determined by:

(4) $$n=\frac{\left(R_{L}+R_{IM}+R_{SW}\right)C_{L}}{T_{clk}}.$$

Since $R_{IM}$ and $R_{SW}$ are fixed resistors, then the time constant of the charge path varies with $R_{L}$ and $C_{L}$. The number of clock pulses (n) is used to calculate the ETI impedance. A greater ETI impedance results in more clocks, whereas a lower ETI impedance results in fewer clocks.

## 5. Estimation of Stimulation Current in IMM Mode

Furthermore, the *n* values are counted by the 3-bit counter where the base value is 2. Once the bit streams become stable, the latch sets to an update. Later, these bits are applied to the current digital to analog (DAC) for further processing. With programmable currents of $I_{2}$, $I_{1}$, and $I_{0}$, the output of current DAC is expressed as:

(5) $$I_{DAC}=b_{2}I_{2}+b_{1}I_{1}+b_{0}I_{0}.$$

**Figure 4:**
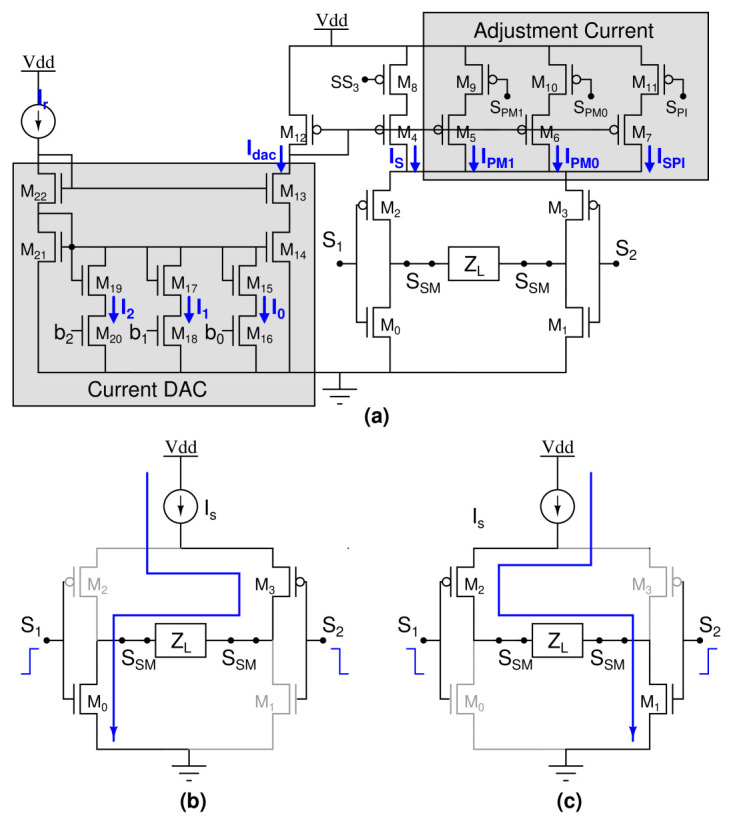
(**a**) Stimulation circuit. (**b**) Cathodic configuration. (**c**) Anodic configuration.

Furthermore, $I_{S}$ is the current mirror of $I_{DAC}$, which is shown in Figure 4.

## 6. Stimulation Mode (SM)

The programmable stimulation circuit consists of an H-structure stimulation circuit, current DAC, and adjustment current sources, as shown in Figure 4a. $M_{0}$-$M_{3}$ forms the H-structure stimulation circuit for shaping the biphasic waveform. The biphasic waveform contains a cathodic pulse and anodic pulse. The cathodic formation of the stimulation circuit generates a negative current to ETI for an action potential as shown in Figure 4b. The anodic formation of the stimulation circuit generates a positive current to ETI so as to neutralize the potential created by the initial cathodic phase as shown in Figure 4c. $M_{13}$-$M_{22}$ forms the current DAC, which has programmable bits $b_{2}$, $b_{1}$, $b_{0}$. Based on the estimated programmable bit in IMM, the stimulation current is applied to the H-structure stimulation circuit. $M_{5}$-$M_{7}$ and $M_{9}$-$M_{11}$ are used to adjust the stimulation current based on residual voltage for charge balancing. $S_{PM1}$, $S_{PM0}$, and $S_{PI}$ are the adjustment switches that are controlled by the adjustment control unit.

## 7. Hybrid Charge Balancing Mode

In general, a negative pulse’s current ($I_{C}$) is usually 1% to 5% higher or lower than a positive pulse’s current ($I_{A}$). This is due to the inevitability of device imperfections during fabrication. The net equivalent current (∆I) is $I_{C}\;I_{A}$. The residual voltage after a single biphasic pulse is $V_{res}=∆IR_{L}$. For *m* stimulation, if the residual voltage is greater than 100 mV, it leads to tissue damage. Thus, it is necessary to maintain the residual voltage within safety limits (<100 mV). This mode’s primary goal is to measure the residual voltage across the load and classify it into 8 levels. Based on these levels, appropriate techniques are applied to balance the charge.

**Figure 5:**
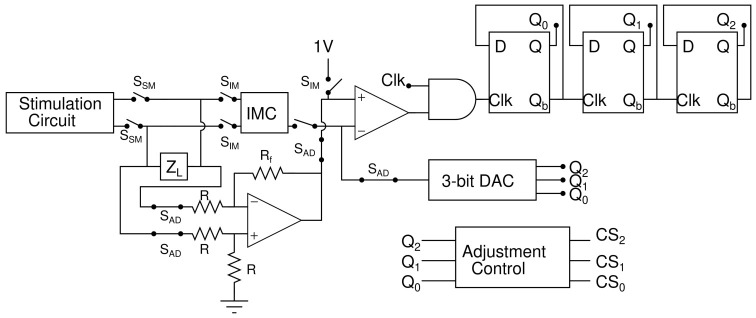
Circuit configuration of hybrid charge balancing mode (HCBM).

The circuit configuration of the hybrid charge balancing mode is shown in Figure 5. In this mode, the $S_{AD}$ switch are turned ON and remaining switches are turned OFF. A voltage subtractor is connected across the $Z_{L}$ to measure residual voltage ($V_{RES}$) which will be further fed to the comparator. On the other hand, the DAC generates eight different voltage levels in ascending order, ranging from −100 mV to 100 mV with a step of 25 mV. The comparator compares the $V_{RES}$ with the DAC voltage ($V_{DAC}$) and feeds the result to one of the AND gate inputs. When $V_{RES}$ is greater than $V_{DAC}$, then the AND gate produces clock pulses to counter. Counter output is fed back to the DAC. When $V_{RES}$ is less than $V_{DAC}$, the AND gate halts generating clock pulses. The counter and DAC are stable. Furthermore, stable counter output is fed to the adjustment control logic. Based on the counter output, the adjustment control unit determines the appropriate technique, as shown in Table 1.

**Figure 6:**
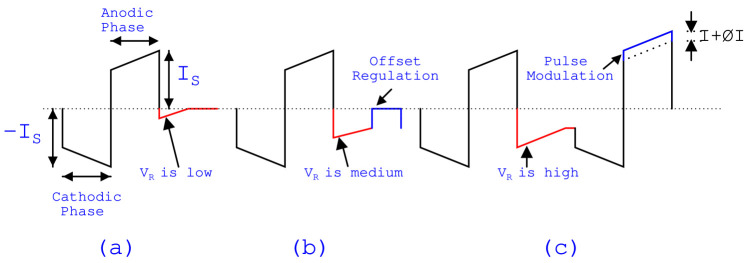
(**a**) When residual voltage is low, (**b**) when residual voltage is medium, and (**c**) when residual voltage is high.

When residual voltage is low, which is near to safety limits, electrodes are shorted for a while to discharge the residual voltage as shown in Figure 6a. When the residual charge is medium, an offset regulation technique with equivalent opposite voltage is used, as shown in Figure 6b. When residual charge is high, then a pulse modulation technique is used where equivalent opposite voltage is applied to the next stimulation waveform, as shown in Figure 6c.

There are three possible cases in which the circuit can adjust the maximum current.

- Case 1—Pulse modulation: In this case, the circuit can adjust a maximum current of 175 µA when SPM1 and SPM0 are turned ON.
- Case 2—Offset regulation: In this case, the circuit can adjust a maximum current of 75 µA when the SPI switch is turned ON.
- Case 3—Electrode shorting: In this case, the circuit will not provide any current adjustment. It can only provide a discharge path for the electrode.

## 8. Adjustment Control Unit

**Figure 7:**
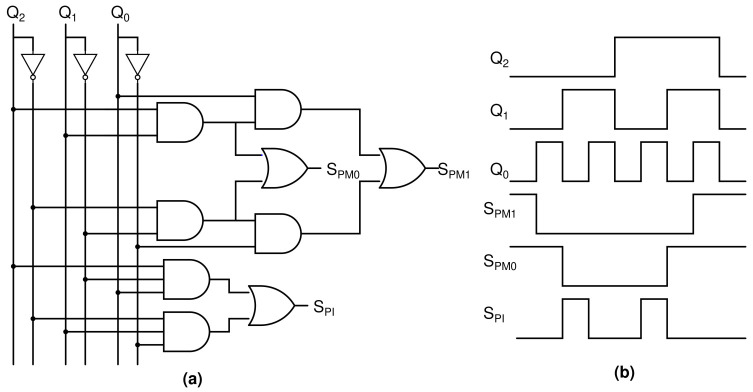
Adjustment control logic (**a**) Circuit configuration and (**b**) Output waveforms.

Figure 7 shows the circuit configuration of the adjustment control logic. The adjustment control unit’s function is to generate appropriate technique control signals from counter outputs. Table 1 depicts the relationship between counter outputs and control signals. When the counter outputs are “011” or “100”, the residual voltage is close to zero, and the electrode shorting technique is used. This technique effectively eliminates low residual voltage by connecting electrodes to ground with no additional power usage. When the counter output is “010” or “101”, it means that the residual voltages are moderately negative or positive, respectively. In this case, a voltage that is approximately equivalent and opposite is applied to the load before the next stimulation pulse. This technique is called offset regulation. When the counter output is “000” or “001”, or “110” or “111”, it means that the residual voltages are high. In this case, an extra current is added to the next stimulation pulse. This technique is called the pulse modulation technique. A logic diagram of the adjustment control signal ($S_{PM1}$, $S_{PM0}$, $S_{PI}$) is derived from counter outputs ($Q_{2}$, $Q_{1}$, $Q_{0}$) using k-map:

(6) $$S_{PM1}=Q_{2}Q_{1}Q_{0}+\bar{Q_{2}}\bar{Q_{1}}\bar{Q_{0}}$$

(7) $$S_{PM0}=Q_{2}Q_{1}+\bar{Q_{2}}\bar{Q_{1}}$$

(8) $$S_{PI}=Q_{2}\bar{Q_{1}}Q_{0}+\bar{Q_{2}}Q_{1}\bar{Q_{0}}.$$

The corresponding logic circuit that generates $S_{PM1}$, $S_{PM0}$, and $S_{PI}$ is shown in Figure 7.

## 9. Results

**Figure 8:**
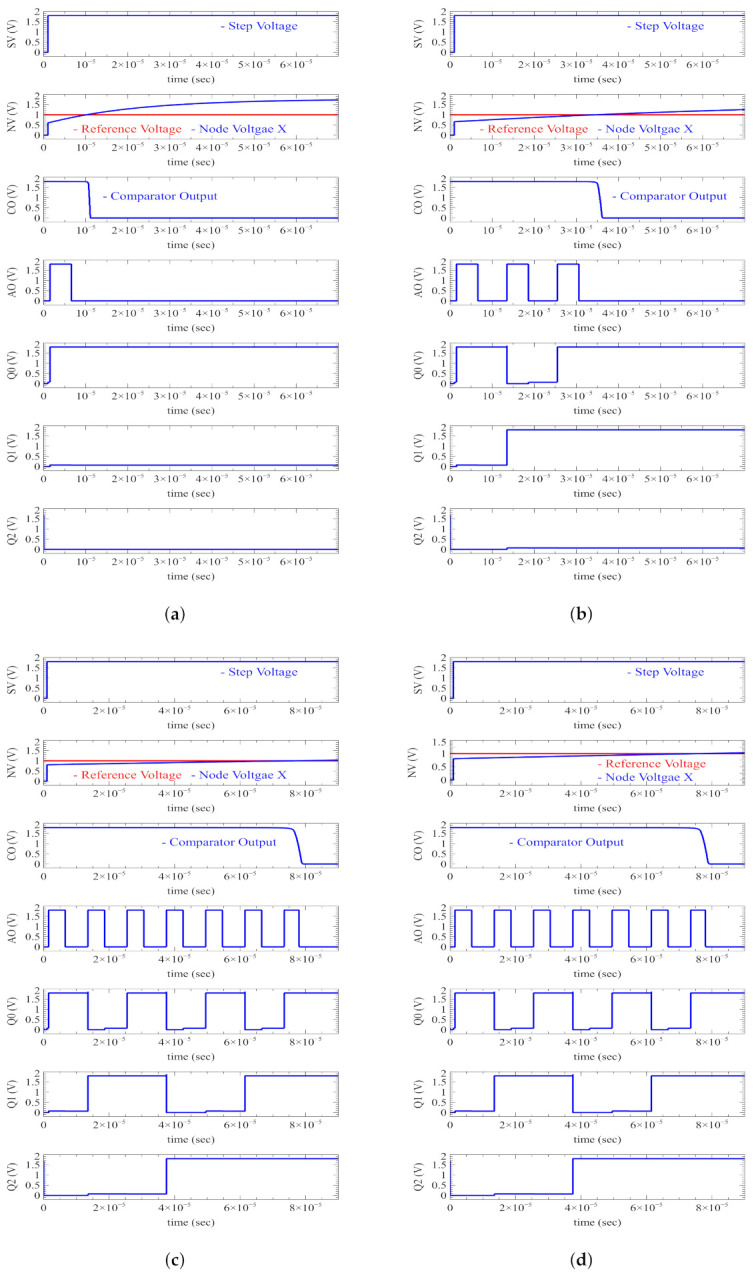
Control bits w.r.t load ($Z_{L}$) variations. (**a**) When the load is 0.2 $Z_{L}$. (**b**) When the load is 0.8 $Z_{L}$. (**c**) When the load is 1.5 $Z_{L}$. (**d**) When the load is 2.6 $Z_{L}$.

The proposed architecture is designed with UMC 180 nm technology. In order to test the proposed design, the ETI load is assumed as a series combination of resistance ($R_{L}$) of 1 kΩ and capacitance ($C_{L}$) of 10 nF [17]. In Figure 8, the output signals of various components in IMM are shown in four cases: (a) when the load is 0.2 $Z_{L}$, (b) when the load is 0.8 $Z_{L}$, (c) when the load is 1.5 $Z_{L}$, and (d) when the load is 2.6 $Z_{L}$. When a 1.8 V step voltage ($V_{IM}$) is applied, the $V_{X}$ voltage gradually rises from 0 V to 1.8 V, depending on $Z_{L}$. The comparator output is high when $V_{X}$ is smaller than $V_{r}$ (1 V), and AND generates clocks. In the case of 0.2 $Z_{L}$, the AND gate generates one clock pulse, as shown in Figure 8a. After the comparator output is low, the counter output ($Q_{2}$, $Q_{1}$, $Q_{0}$) is fed to the current DAC, where it is (0, 0, 1), as shown in Figure 8a. In the case of 0.2 $Z_{L}$, the counter output is (0, 0, 1), which indicates less than the intended value, thus stimulation current is reduced and driven to the load. Similarly, for 0.8 $Z_{L}$ 3 clock pulses are applied to the counter as shown in Figure 8b. As a result, the stimulation current is reduced and driven to the load. In the case of 1.5 $Z_{L}$ and 2.6 $Z_{L}$, the counter outputs are (1, 0, 1) and (1, 1, 1), indicating that the value is greater than the intended value as shown in Figure 8c and Figure 8d, respectively. Therefore, the stimulation current is raised and sent to the load.

**Figure 9:**
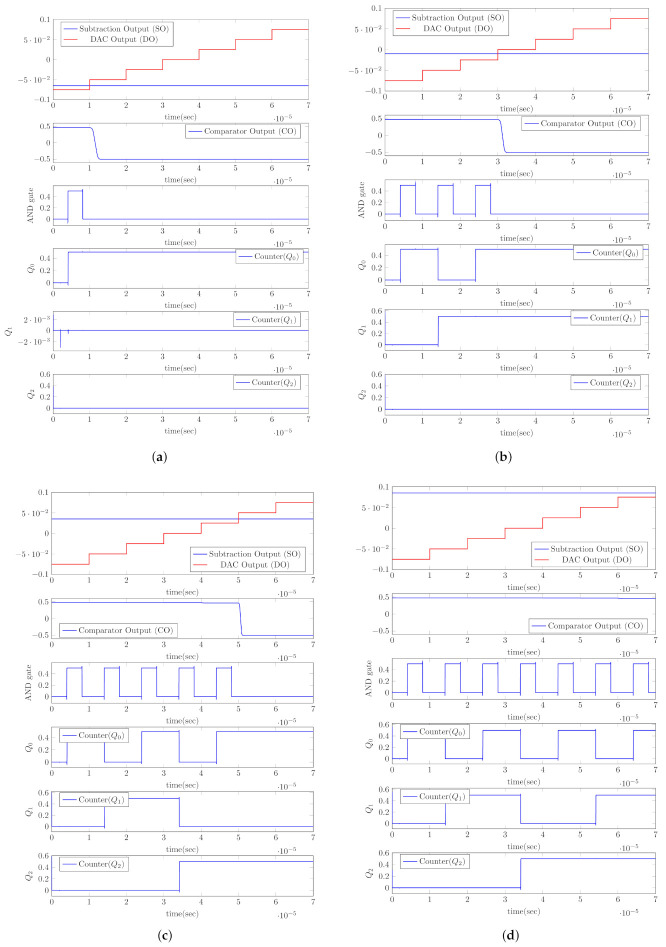
Calibration output waveform when residual voltage is positive. (**a**) When −75 mV < V_r_<−50 mV. (**b**) When −25 mV < V_r_< 0 mV. (**c**) When 25 V < V_r_< 50 mV. (**d**) When V_r_> 75 mV.

Two negative residual voltages of −65 mV and −10 mV, as well as two positive residual voltages of 35 mV and 85 mV, are employed to verify the operation of the proposed calibration circuit, as illustrated in Figure 9. The subtractor output ($V_{S}$) is between −75 mV and −50 mV in the circumstance presented in Figure 9a, causing the comparator to fall from high to low at the 2nd level comparison. As a result, the AND gate’s output is 1 pulse. Finally, the counter counts the pulses and outputs (0, 0, 1) to the stimulation circuit via the logic circuit (Q2, Q1, Q0). Similarly, the subtractor output ($V_{S}$) in this Figure 9b scenario is between −25 mV and 0 mV, causing the comparator to fall from high to low at the 4th level comparison. As a result, the AND gate’s output is three pulses. Finally, the counter counts the pulses and outputs (0, 1, 1) to the stimulation circuit via the logic circuit (Q2, Q1, Q0). The subtractor output ($V_{S}$) in this Figure 9c situation is between 25 mV and 50 mV, causing the comparator to fall from high to low at the 6th level comparison. As a result, the AND gate’s output is five pulses. Finally, the counter counts the pulses, and its output (1, 0, 1) is fed back to the stimulation circuit via the logic circuit (Q2, Q1, Q0). The subtractor output ($V_{S}$) in this Figure 9d situation is greater than 75 mV, causing the comparator to remain high. As a result, the AND gate’s output is 7 pulses. Finally, the counter counts the pulses and outputs (1, 1, 1) to the stimulation circuit via the logic circuit (Q2, Q1, Q0).

**Figure 10:**
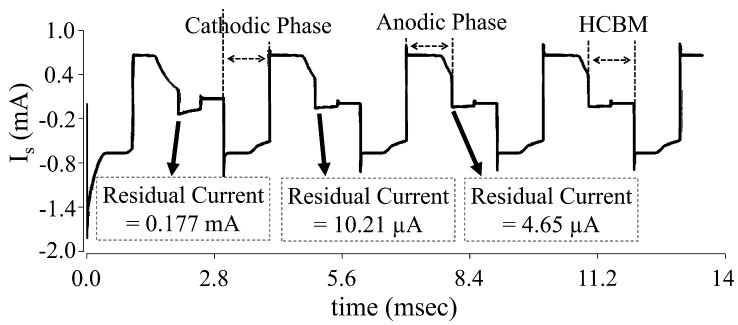
Stimulation current across load.

In all operating modes, Figure 10 depicts the output current flowing through the load. Due to the imbalance in the first stimulation waveform, the maximum and minimum residual current ($I_{res})$ is 177 µA and 90 µA, respectively. The residual voltage ($V_{res}$) across the load resistor after the first biphasic pulse is $V_{res}$ = $I_{res}$ $R_{L}$ = 90 mV. According to [18], the residual voltage should not exceed 100 mV, which may lead to tissue damage. Therefore, in order to avoid this situation, the proposed circuit switches to an offset regulation technique, which adds current (75 μA) in the opposite direction. As a result, the residual current is reduced to 10.21 μA in the next biphasic pulse. The proposed circuit switches to an electrode shorting technique as the residual current is a low value (10.21 μA). Similarly, the residual current in the next biphasic pulse is 4.65 μA, which is achieved by using the electrode shorting technique once again.

**Figure 11:**
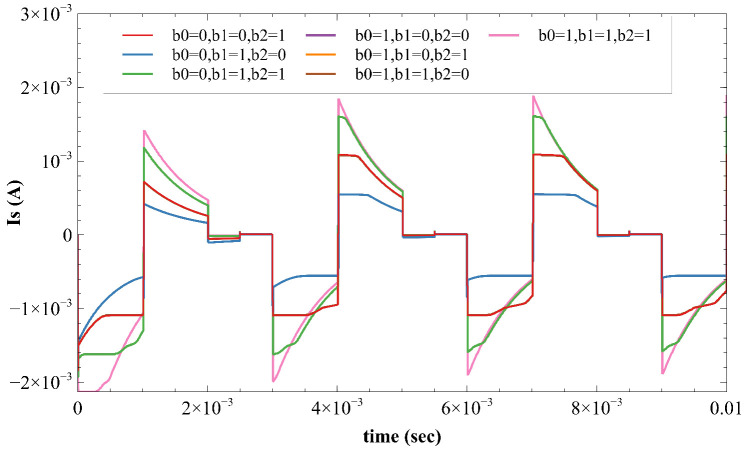
Different stimulation current with respective programmable bits.

The stimulation current varies between 0.8 mA and 1.4 mA as shown in Figure 11, which leads to a maximum power consumption of 2.52 mW. The maximum power consumption of IMM and HCBM is 44 μW. Then there is a power ratio of 1.74 percent to 5.5 percent, which is the ratio of the suggested calibration circuit to the overall stimulation circuit.

**Figure 12:**
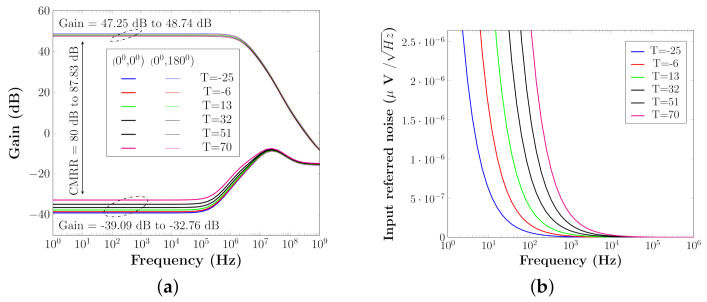
Operational amplifier (Op-Amp) characteristics. (**a**) Op-Amp characteristics respective to temperature. (**b**) Op-Amp noise response respective to temperature.

The AC analysis of Operational amplifier (Op-Amp) is shown in Figure 12a. The pre-amplifier (PA) delivers a gain of 48.74 dB, according to the results. PA has a common mode rejection ratio of more than 80 dB, which is suitable for the calibration design. At 100 Hz–100 KHz, PA’s input referred noise is less than 0.1 $\mu V_{rms}$/sqrt(Hz) as shown in Figure 12b.

## 10. Conclusions

The proposed architecture achieves optimal performance by addressing both the issues of load impedance variation and charge imbalances. Table 2 compares the proposed hybrid charge balancing circuit to recently published designs that use various active charge balancing approaches. In comparison to the state of the art, this comparison shows that the hybrid charge balancing circuit with adjustable ETI impedance can give maximum energy efficiency. This is due to the fact that HCBM is a hybrid of electrode shorting, offset regulation, and pulse modulation, which combines the best features of each technique and applies them to appropriate situations. In addition, the IMM measures ETI to achieve the desired stimulation currents. The results show that the proposed IMM and HCBM configurations have the most efficient power use.

## 11. Patents

- Ganesh Lakshamana Kumar Moganti, V. N. Siva Praneeth, and Siva Rama Krishna Vanjari, “A Hybrid Charge Balancing Technique with Adaptive ETI Impedance Variations for Facial Paralysis Patients”, in Official Journal of The Patent Office, Indian Patent Office, filed and published on 10 September 2021.
- Ganesh Lakshamana Kumar Moganti, V. N. Siva Praneeth, and Siva Rama Krishna Vanjari “An Implantable Bipolar Active Charge Balancing Circuit with Six Adjustment Current levels for Facial Paralysis Patients”, in Official Journal of The Patent Office, Indian Patent Office, filed and published on 23 July 2021.

## Figures and Tables

**Table 1:** Adjustment control unit characteristics.

$Q_{2}$	$Q_{1}$	$Q_{0}$	$S_{\mathrm{pm}1}$	$S_{\mathrm{pm}0}$	$S_{\mathrm{pi}}$	Technique
0	0	0	ON	ON	OFF	Anodic pulse modulation
0	0	1	OFF	ON	OFF	Anodic pulse modulation
0	1	0	OFF	OFF	ON	Offset regulation
0	1	1	OFF	OFF	OFF	Electrode shorting
1	0	0	OFF	OFF	OFF	Electrode shorting
1	0	1	OFF	OFF	ON	Offset regulation
1	1	0	OFF	ON	OFF	Cathodic pulse modulation
1	1	1	ON	ON	OFF	Cathodic pulse modulation

**Table 2:** Similar works.

	This Work	IEEE Access 2020 [15]	IEECON 2020 [19]	TBioCAS 2018 [12]	TBioCAS 2016 [20]	TBioCAS 2015 [14]	TBioCAS 2015 [13]
Technology	0.18 μm	0.18 μm	0.35 μm	0.6 μm	0.18 μm	0.18 μm	0.18 μm
Stimulation Current	0.8 mA–1.4 mA	1	4 μA–1 mA	0.095	upto 3 mA	10 mA	1.45 mA
Current resolution	3-bit	5-bit	5-bit	8-bit	15-bit	6-bit	9-bit
Voltage	1.8 V	12.3 V	20V	12 V	12 V	20 V	12 V
Technique	RVM + BPM	ACM + ES	OR	ES	ES	OR	DBC
Charge Balance	Bipolar	Mono polar	-	-	Bipolar	Mono polar	-
ETI variations	Yes	No	No	No	No	No	No

RVM: Residual voltage measure, BPM: Bipolar modulation, ES: Electrode shorting, ACM: Anodic current modulation, OR: Offset regulation, and DBC: DC blocking capacitor.

## Data Availability

Not applicable.

## Abbreviations

The following abbreviations are used in this manuscript:

ETI	Electrode Tissue Interface
FES	Functional Electrical Stimulation
IMM	Impedance Measurement Mode
HCBM	Hybrid Charge Balancing Mode
SM	Stimulation Mode
DAC	Digital to Analog Converter
RVM	Residual Voltage Measure
BPM	Bipolar Modulation
ES	Electrode Shorting
ACM	Anodic Current Modulation
OR	Offset Regulation
DBC	DC Blocking Capacitor
PA	Pre-amplifier
